# Regulatory T cells in lung disease and transplantation

**DOI:** 10.1042/BSR20231331

**Published:** 2023-10-27

**Authors:** Peizhen Lao, Jingyi Chen, Longqian Tang, Jiwen Zhang, Yuxi Chen, Yuyin Fang, Xingliang Fan

**Affiliations:** Institute of Biological and Food Engineering, Guangdong University of Education, 351 Xingang Middle Road, Guangzhou 510303, PR China

**Keywords:** Foxp3, Immunosuppression, Lung disease, Regulatory T cells, TGF-β

## Abstract

Pulmonary disease can refer to the disease of the lung itself or the pulmonary manifestations of systemic diseases, which are often connected to the malfunction of the immune system. Regulatory T (Treg) cells have been shown to be important in maintaining immune homeostasis and preventing inflammatory damage, including lung diseases. Given the increasing amount of evidence linking Treg cells to various pulmonary conditions, Treg cells might serve as a therapeutic strategy for the treatment of lung diseases and potentially promote lung transplant tolerance. The most potent and well-defined Treg cells are Foxp3-expressing CD4^+^ Treg cells, which contribute to the prevention of autoimmune lung diseases and the promotion of lung transplant rejection. The protective mechanisms of Treg cells in lung disease and transplantation involve multiple immune suppression mechanisms. This review summarizes the development, phenotype and function of CD4^+^Foxp3^+^ Treg cells. Then, we focus on the therapeutic potential of Treg cells in preventing lung disease and limiting lung transplant rejection. Furthermore, we discussed the possibility of Treg cell utilization in clinical applications. This will provide an overview of current research advances in Treg cells and their relevant application in clinics.

## Introduction

Pulmonary disease can refer to either the lung disease or the respiratory symptoms of systemic diseases. There are different kinds of lung diseases in human beings, the most common type of pulmonary disease is bronchitis, which is an inflammation of the air passages. Pulmonary disease can be caused by a variety of factors, including infection, exposure to toxins or irritants and smoking. According to the aetiology, pulmonary diseases can be categorized into immune-related lung diseases (i.e. bronchial asthma and allergic rhinitis), lung diseases caused by air pollution and smoking (i.e. chronic obstructive pulmonary disease, which encompasses obstructive emphysema and chronic bronchitis), lung tumors, infectious lung diseases (such as pneumonia, tuberculosis, etc.), lung manifestations of systemic diseases, etc [1].

The lung contains the highest density of blood vessels in the human body, which link the respiratory system to the outside world [2]. It would cause serious damage and even be lethal if a human lung disease manifests. Treatment for pulmonary disease depends on the specific type and severity of the condition. In some cases, medication including oral, intravenous drip, and inhalation therapy may be necessary to manage symptoms [3]. In other cases, surgery may be necessary to remove damaged tissue or correct a problem with lung transplantation when appropriate.

It has become increasingly apparent that lung disorders are often connected to the malfunction of the immune system [4,5]. Regulatory T (Treg) cells have an important role in maintaining immune balance in the body through immune regulation and immunosuppression. Treg cells are a subpopulation of CD4^+^ T cells that are critical for maintaining self-tolerance and preventing autoimmune disease [6,7]. Treg cells were initially characterized as CD4^+^CD25^+^ T cells, which constitute 5–10% of peripheral CD4^+^ T cells in humans [8]. Furthermore, Foxp3 has been identified as the main transcription factor that distinguishes this lineage of thymic Treg cells [8,9].

There is growing evidence that Treg cells are involved in the development and progression of a number of different lung disorders [10,11]. For example, it has been shown that Treg cells are inappropriately activated in the lungs of patients with asthma and chronic obstructive pulmonary disease (COPD) [11–13]. In addition, Treg cells are deficient in patients with idiopathic pulmonary fibrosis (IPF) [14,15]. However, Treg cells have been shown a controversial role in cancer progression. Treg cell infiltration into tumors is a contributor to poor prognosis via hindering effective immune response against tumor cells in non-small cell lung cancer (NSCLC) [16,17].

Given the growing body of evidence linking Treg cells to various lung disorders, Treg cell function manipulation might be a potential therapeutic strategy for treating lung disorders. Moreover, the expanded role of Treg cells in inflammation regulation serves as a rationale for methods to increase Treg cell populations [18]. Therefore, it is hoped that a better understanding of the role of Treg cells in lung disease will lead to more effective treatments for these conditions. In this review, we summarize the characteristics of Treg cells and discuss the regulatory role of Treg cells against lung diseases and transplant rejection. We have summarized the mechanisms of action and therapeutic studies of Treg cells in lung diseases to provide readers with a clearer and more structured overview of the content (Table 1). This will provide an overview of current research advances in Treg cells and their relevant application in clinics.

**Table 1:** The mechanisms of action and therapeutic studies of Treg cells in lung diseases

Disease	Type of Treg cells	Main way	Main profile	Outcome	Reference
Asthma	CD4^+^ Treg cells, Foxp3^+^ Treg cells	TGF-β, IL-10, DLL4-Notch signalling pathway	↓ ILC2, Th1 cells, Th2 cells, Th17 cells, Granulocytes	Inhibition	[153,158–162]
ARDS	Foxp3^+^ Treg cells	TGF-β, IL-10	↓ Lung pyroptosis, Th17 cells	Inhibition	[168,170,171,175]
IPF	CD4^+^Foxp3^+^ Treg cells	TGF-β	↑ TGF-β1	Early: Promotion	[174,181]
			↓ Th17 cells, CXCL10, Fibrosis	Late stage: Inhibition	
COPD	Foxp3^+^ Treg cells	TGF-β, IL-10	↓ HIF-1α, TNF-α, IL-1β, IL-17A, RORγt	Inhibition	[13,34,169,184]
CF	Foxp3^+^ Treg cells	TGF-β, IL-10	↓ IL-6, IL-8, IL-17A	Inhibition	[186,193]
Lung cancer	CD4^+^Foxp3^+^ Treg cells, CD25^+^ Treg cells	TGF-β, IL-10	↑ HIF-1α, CTLA4, CD39, GITR, B7H1, IFN-γ, CXCL13	Promotion	[17,199,202–204,208–210,212–215]
			↓ CD8^+^ T cells, NK cells, IL-2		
Bronchitis	Nrp-1^+^ Treg cells	TGF-β, IL-10	↑ IL-35, Glentiy-1	Inhibition	[161,235,236,322–325]
			↓ IL-17, TIM-3, Macrophages, MHC class II molecules		
Pneumonia	Foxp3^+^CD39^+^ Treg cells	TGF-β, IL-10, IL-35	↑ EGF, BLIMP1	Inhibition	[240,243,245,247,252,258]
			↓ CD4^+^ and CD8^+^ T cells, B cells, DCs, NK cells, Neutrophils, Eosinophils		
Pulmonary embolus	CD4^+^Foxp3^+^ Treg cells	TGF-β	↑ SPARC, Monocytes	Inhibition	[264]

Abbreviations: ARDS, acute respiratory distress syndrome; BCL6, B-cell lymphoma 6; BLIMP1, B-lymphocyte-induced maturation protein 1; CF, cystic fibrosis; COPD, chronic obstructive pulmonary disease; CTLA4, cytotoxic T-lymphocyte-associated protein 4; CXCL10, C-X-C motif chemokine ligand 10; CXCL13, C-X-C motif chemokine ligand 13; DC, dendritic cells; DLL4, delta-like 4; EGF, epidermal growth factor; GITR, glucocorticoid-induced tumour necrosis factor receptor; HIF-1α, hypoxia-inducible factor 1α; IFN-γ, interferon-γ; IL-1β, interleukin-1β; IL-10, interleukin-10; IL-17A, interleukin 17A; IL-35, interleukin-35; ILC2, Type 2 innate lymphocytes; IPF, idiopathic pulmonary fibrosis; MHC, major histocompatibility complex; NK, natural killer; SPARC, scalable processor architecture; TGF-β, transforming growth factor β; Th1, Type 1 T helper; Th2, Type 2 T helper; Th17, Type 17 T helper; TIM-3, T-cell immunoglobulin and mucin domain-containing protein 3; TNF-α, tumor necrosis factor alpha.

## Biology of Treg cells

### Origin and development of Treg cells

Treg cells are a specialized subset of T cells that play an indispensable role in maintaining the body’s immune homeostasis and preventing autoimmunity. These cells are characterized as CD4^+^Foxp3^+^ Treg cells [19]. Foxp3, discovered as a marker molecule of Treg cells, is a member of the forkhead transcription factor family, which plays a crucial role in the immune regulation of the human body [20,21]. Foxp3 mutation can hinder the development and normal function of Treg cells, resulting in the development of immune dysregulation and autoimmune diseases [22–26]. Apart from its role as a marker molecule of Treg cells, Foxp3 also serves as a regulatory factor that can modulate the activity of Treg cells by regulating other genes including *Il2, Ifng* and *Ctla4* [23,26].

**Figure 1:**
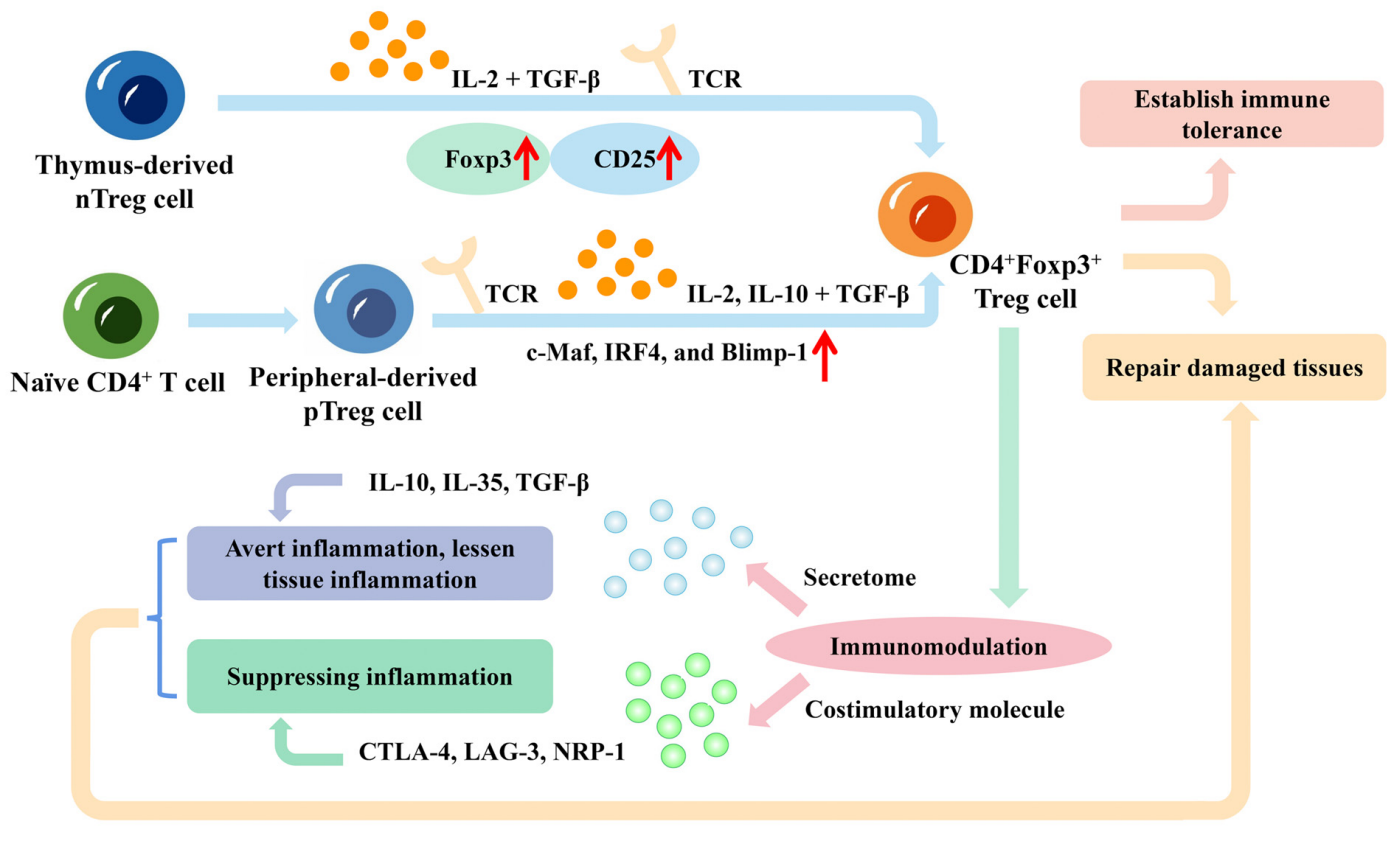
The origin and function of CD4^+^Foxp3^+^ regulatory T (Treg) cells Thymus-derived regulatory cells (tTreg cells), which express high levels of CD25 and forkhead box protein P3 (Foxp3), develop in the thymus from precursors that were born in the bone marrow. These cells migrate to the thymus and differentiate into CD4^+^Foxp3^+^ Treg cells. The differentiation of tTreg cells is driven by signals mediated by the T-cell receptor (TCR) and by cytokines such as interleukin (IL)-2 and transforming growth factor-beta (TGF-β). The peripheral original peripherally-derived Treg cells (pTreg cells) are induced or generated from naïve CD4^+^ T cells in response to the combination of TCR signalling and exposure to TGF-β as well as IL-2 and IL-10. Transcription factors such as c-Maf, interferon regulatory factor 4 (IRF4), and B-lymphocyte-induced maturation protein 1 (Blimp-1) also play important roles in pTreg cell differentiation. Effector Treg cells have been implicated in immunomodulation, tissue repair, and immune tolerance. Treg cells can act as an immunomodulator via cytokines and cell-cell contact. These cells can generate anti-inflammatory factors IL-10, IL-35 and TGF-β to avert inflammation and lessen tissue inflammation. Treg cells have inhibitory molecules, for instance, cytotoxic T-lymphocyte-associated protein 4 (CTLA-4), lymphocyte activation gene 3 (LAG-3) and neuropilin-1 (NRP-1), which aid in suppressing inflammation.

CD4^+^Foxp3^+^ Treg cells can be classified into two major types based on their developmental origins: thymus-derived Treg cells (tTreg cells) and peripherally-derived Treg cells (pTreg cells) [24,27]. As shown in Figure 1, the majority of CD4^+^Foxp3^+^ Treg cells develop in the thymus from precursors that were born in the bone marrow. These cells migrate to the thymus, where they recognize self-antigens presented by thymic epithelial cells, ultimately differentiating into CD4^+^Foxp3^+^ Treg cells [28,29]. The thymus-generated Treg cells are also referred to as naturally developed Treg cells (nTreg cells), which express high levels of CD25 and Foxp3 [28–30]. The expression of Foxp3 is initiated during development in the thymus but is not yet stable. The long-term persistence and differentiation of this type of tTreg cell in peripheral tissues are largely dependent on the assistance of TGF-β [28,31–34]. The differentiation of tTreg cells is driven by signals mediated by the T-cell receptor (TCR) and by cytokines such as interleukin (IL)-2 and transforming growth factor-β (TGF-β) [35]. Once they mature, tTreg cells migrate to the periphery where they can suppress the activation of autoreactive T cells.

The peripheral original pTreg cells are induced or generated from naïve CD4^+^ T cells in response to antigenic stimulation. Their differentiation is induced by the combination of TCR signalling and exposure to TGF-β and other cytokines, such as IL-2 and IL-10 [26,27,36]. These cytokines activate downstream signalling pathways that lead to the up-regulation of Foxp3. Foxp3-transformed T cells require T-cell receptor (TCR) stimulation to exert inhibitory effects. In addition, other transcription factors such as c-Maf, IRF4, and Blimp-1 also play important roles in pTreg cell differentiation [37]. When expressed, Foxp3 will also promote the development and function of Treg cells in a CD28-dependent manner, which can be further stimulated by TGF-β for increased Foxp3 expression in Treg cells [36,38–40]. It can be concluded that TGF-β has a crucial role in inducing the development of Foxp3^+^ Treg cells (Figure 1). pTreg cells can arise from multiple subsets of peripheral CD4^+^ T cells, including naïve T cells, memory T cells, and even type 1 T helper (Th1) and type 17 T helper (Th17) cells, etc [41].

Both tTreg and pTreg cells are important for maintaining immune tolerance and preventing autoimmunity [42]. These CD4^+^Foxp3^+^ Treg cells can modulate the balance and stability of the immune system within the body by regulating the timing and magnitude of immune responses, not just to autoimmunity but also to other antigens [42–45]. The CD4^+^Foxp3^+^ Treg cells not only have a vital role in immune regulation but also serve as a benchmark for disease diagnosis and treatment. When a disease manifests, the proportion of CD4^+^Foxp3^+^ Treg cells decreases, and the expression of Foxp3 is reduced [46]. Furthermore, augmenting the expression of Foxp3 or other pathways *in vivo* may increase the number of CD4^+^Foxp3^+^ Treg cells, resulting in better therapeutic results [47–49].

### Effector CD4^+^Foxp3^+^ Treg cells

The effector CD4^+^Foxp3^+^ Treg cells not only possess a regulatory function but also exhibit certain effector functions, such as the ability to secrete cytokines or migrate to inflamed tissues. Effector CD4^+^Foxp3^+^ Treg cells have been implicated in a variety of immune processes, including immunomodulation, repairing damaged tissues, establishing immune tolerance, etc (Figure 1). They can suppress the immune response of innate and adaptive immune cells, including conventional T cells, B cells and natural killer (NK) cells. In general, Treg cells exert their immunomodulatory effects on various types of immune cells through three mechanisms: soluble factor mediation (such as anti-inflammatory cytokines, FLG-2, adenosine, granzyme and perforin), inhibitory receptor engagement and growth factor competition [50,51]. These three approaches often work in conjunction, thereby strengthening the immune regulation of Treg cells.

Specifically, the Foxp3^+^ Treg cell can secrete different types of cytokines (IL-10, IL-35 and TGF-β) to suppress immune activation, particularly in instances where inflammation takes place in an organ or tissue. IL-10 can suppress the activation of antigen-presenting cells (APCs) by inhibiting CD28 tyrosine phosphorylation, leading to a decrease in the immune response of CD4^+^ T cells [52]. Furthermore, IL-10 can inhibit effector T cells and B cells, as well as down-regulate major histocompatibility complex class I (MHC-I) expression, resulting in reduced inflammation [53]. Similarly, TGF-β is a multi-functional cytokine with potent anti-inflammatory properties that inhibits the differentiation of effector T cells, thereby exerting immunosuppressive effects [54–56]. IL-35, on the other hand, induces the proliferation of CD4^+^CD25^+^ regulatory T cells and inhibits Th17 differentiation [57]. Although it can be produced by Treg cells through strong stimulation, IL-35 efficacy in humans is still limited [57]. Treg cells can also secrete FLG-2, adenosine, granzyme A/B and perforin to exert immunosuppressive effects. FLG-2 can hinder T-cell polarization toward Th2 and simultaneously down-regulate Th1 and Th17 immune responses [58]. It can also inhibit dendritic cell (DC) maturation by reducing the expression of CD80 and MHC II molecules [59], and binding to the FcγRIIB receptor expressed by APCs to exert an inhibitory effect [60]. Cells surrounded by adenosine can facilitate the transformation of pro-inflammatory immune cells that are activated by ATP into anti-inflammatory cells through CD39 and CD73, thereby reversing the pro-inflammatory environment [61]. Granzyme A/B and perforin interaction rely on contact with effector cells to function. Perforin can form transportive perforin channels after contacting effector cell membranes, while granzyme A/B can enter effector cells either through perforin channels or via mannose-6-phosphate receptor-dependent mechanisms [53,62]. This leads to the cleavage of caspases and activation of intracellular enzymes, which results in apoptosis of effector cells and immune response inhibition [63,64]. Granzyme B is highly expressed by Treg cells in tumor diseases, and it can induce apoptosis of NK cells and CD8^+^ T cells [53,65]. Moreover, studies have shown that granzyme B can help build tolerance in transplants [66].

Treg cells employ inhibitory receptors including cytotoxic T lymphocyte-associated protein 4 (CTLA-4), neuropilin-1 (NRP-1), Galectin-1, LAG-3 and TIM-3 to exert immunosuppressive effects, which aid in suppressing inflammation [67]. CTLA-4 is constitutively expressed on Treg cells and can effectively suppress autoreactive T cells [68]. CTLA-4 and CD28 are cognate receptors that bind to the B7 molecule, but CTLA-4 has a higher affinity for B7 than CD28 [69,70], leading to the inhibition of T-cell activation via arresting the cell cycle, transmitting inhibitory signals to slow down the secretion of IL-2 and decreasing the expression of B7 molecules [71,72]. NRP-1 mediates the formation of an immune synapse between T cells and DCs, limiting the access of effector T cells to APCs and promoting the interaction between Treg cells and naïve DCs [73,74]. Galectin-1 is up-regulated upon Treg activation and, when combined with specific cell surface receptors (such as CD2, CD3, CD4, CD7 and CD43), can block the secretion of pro-inflammatory cytokines (IL-2 and IFN-γ) and enhance the secretion of anti-inflammatory cytokine IL-10, which will induce cycle arrest and enhance apoptosis of activated T cells [75–77]. LAG-3, a homologue of CD4, has a stronger affinity for MHC class II molecules, which can inhibit DC maturation and immune stimulation [78,79]. Such inhibition will further inhibit the activation of T cells. At present, little is known about TIM-3, but its existence has been detected in Treg cells. TIM-3^+^ Treg cells can suppress effector Th17 cells and show high expression of IL-10, CTLA-4, LAG-3 and other factors [80]. Targeted therapy using TIM-3^+^ Treg cells has potential in cancer treatment [81].

Treg cells can exert immunosuppressive effects by engaging in competition for growth factors, with IL-2 being the primary growth factor involved. IL-2 plays a crucial role in the development, survival, and function of Treg cells, as well as in the activation of T cells [82–87]. Thus, competition arises between Treg cells and effector T cells for IL-2. Treg cells express CD25 and form a high-affinity IL-2 trimeric receptor, enabling them to compete with effector T cells for IL-2 in the local environment [88]. If effector T cells lack access to IL-2, they will be unable to proliferate and undergo apoptosis [89].

The immunomodulatory effect of Foxp3^+^ Treg cells can be utilized in treating human autoimmune diseases, for some autoimmune disorders can lead to allergic reactions caused by hypersensitivity to antigens [90]. Apart from their crucial role in regulating the immune system, Foxp3^+^ Treg cells have been found to help repair and heal various tissues such as muscle, bone, lung tissue, heart, kidney tissue, and more [91–107]. When tissue damage occurs, Treg cells can produce anti-inflammatory factors like IL-10 to combat inflammation, as well as secrete growth factors such as amphiregulin and keratinocyte growth factor (KGF) to promote tissue repair and regeneration. Treg cells can also inhibit the function of INF-γ and use IL-33 to regenerate Treg cells and increase their numbers, speeding up the repair process. They can induce macrophage differentiation and suppress adverse cell factors through cell contact and other methods. Furthermore, de Candia et al. found that Treg cells can act as critical metabolic sensors controlling the immune system, by which to restore organismal homeostasis in metabolic disorders [108]. However, the immunosuppressive nature of these cells can create conditions that lead to immune escape, as seen in cancer where they aid cancer cells in evading the immune system. Consequently, cancer cells can proliferate rapidly leading to uncontrolled growth and progression of the disease [109–111].

### Lung Treg cells

Lung-specific Treg cells are specialized to function within the lungs. Different subtypes of regulatory T cells including CD4^+^CD25^+^ Treg cells, Foxp3^+^ Treg cells and type 1 regulatory T cells (Tr1 cells, which secret high levels of IL-10 but lack the expression of Foxp3), pTreg cells play an important role in maintaining immune tolerance in the respiratory system by suppressing inflammation and preventing tissue damage [112,113]. They achieve this by releasing anti-inflammatory cytokines and by directly inhibiting the activity of other immune cells. IL-10 secreted by lung Treg cells and Tr1 cells can inhibit the production of pro-inflammatory cytokines IL-5 and IL-13, which play important roles in respiratory diseases such as asthma. Lung Treg cells can inhibit the activation of type 2 innate lymphocytes (ILC2s) via direct inducible costimulator (ICOS) induction, by which to suppress the production of IL-5 and IL-13 [114,115]. Moreover, lung Treg cells can secrete amphiregulin, a growth factor that stimulates the proliferation and differentiation of epithelial cells and helps to repair damaged lung tissue [99,116,117]. Faustino et al. found that ST2^+^ Treg cells in the lung can act as a negative regulator of early innate γδT cell response by elevating Ebi3, by which to reduce allergen-inducing inflammation in the lung [118]. During acute immune rejection after lung transplantation, there is an increase in the number of Treg cells, which function as immune suppressors. Their role is to decrease immune rejection, thereby contributing to the successful integration of the transplanted lung tissue and enabling transplant recipients to live in harmony with their new lungs [119,120].

### Conversion between Treg cells and other CD4^+^ T-cell subsets

**Figure 2:**
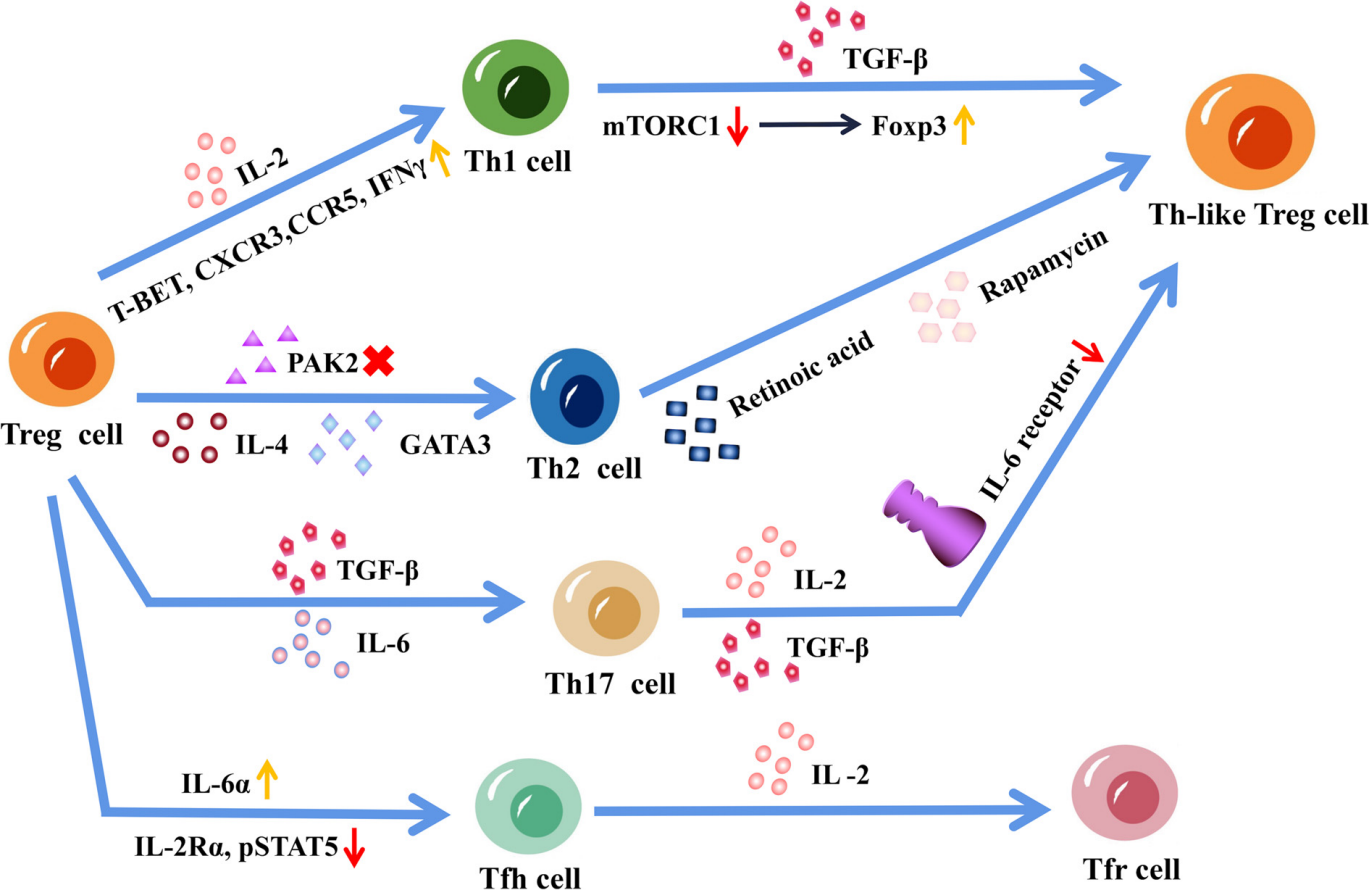
Conversion between regulatory T (Treg) cells and other CD4+ T-cell subsets Under interleukin (IL)-2 stimulation, the expression level of T-BET, C-X-C Motif Chemokine Receptor 3 (CXCR3), C-C Motif Chemokine Receptor 5 (CCR5) and interferon-γ (IFN-γ) was up-regulated, and Treg cells were transformed into type 1 T helper (Th1) cells. In the presence of transforming growth factor-beta (TGF-β), the reduction of mammalian target of rapamycin complex 1 (mTORC1) promoted forkhead box protein P3 (Foxp3) expression and the transformation of Th1 cells into Th1-like Treg cells. IL-4 promotes the conversion of Treg cells to type 2 T helper (Th2) cells, and when p21-activated kinase 2 (Pak2) cannot be expressed, Treg cells express GATA binding protein 3 (GATA3) and Th2-related factors, thereby converting Treg cells into Th2 cells. Under all-trans-retinoic acid and rapamycin stimulation, memory Th2 cells can be transferred to Foxp3^+^ T cells via TGF-β. IL-6 and TGF-β can induce the transformation of naturally developed treg (nTreg) cells to type 17 T helper (Th17) cells, and IL-2 and TGF-β down-regulate IL-6 receptor expression in Treg cells, which are resistant to the transformation of Treg cells into Th17 cells. Under the influence of IL-6α, IL-12, and IL-21, Treg cells can be transferred into T follicular helper (Tfh) cells. This transformation not only induces the expression of IL-6α but also leads to a reduction in the expression of IL-2Rα and phosphorylated STAT-5. IL-2 can promote the transformation of Tfh cells into follicular Treg (Tfr) cells.

Treg cell is one of the subsets of CD4^+^ T cells, which can convert to each other under specific conditions. The conversion between Treg cells and other CD4^+^ T cell subsets is a complex and dynamic process that can occur in response to various environmental signals. Under certain conditions, Treg cells can convert into other CD4^+^ T cell subsets such as Th1, type 2 T helper (Th2), Th17 or T follicular helper (Tfh) cells. Conversely, other CD4^+^ T cell subsets can convert into Treg cells (Figure 2). This conversion is driven by changes in the expression of transcription factors such as Foxp3, T-bet, GATA3, RORγt and BCL6, which are critical regulators of T-cell differentiation [121]. The conversion between Treg cells and other CD4 T-cell subsets plays an important role in maintaining immune homeostasis and can have significant implications for the development of homeostasis in the immune system. However, the mechanisms underlying Treg cell conversion and the factors that regulate this process are still not fully understood and require further investigation.

In humans, stimulating Treg cells with IL-12 will result in the up-regulation of T-BET, C-X-C Motif Chemokine Receptor (CXCR)3, C-C Motif Chemokine Receptor (CCR)5 and interferon-γ (IFN-γ), which induces a Th1-like Treg phenotype via up-regulating the expression [122,123]. Koch et al. found that activation of signal transducer and activator of transcription (STAT)-1 by effector T cell-derived IFN-γ induced T-bet and CXCR3 expression in Treg cells. However, this conversion was abortive due to delayed induction of IL-12 receptor component IL-12 Rβ, which prevented Treg cells from completing STAT-4-dependent Th1 cell differentiation in acute type 1 inflammatory response [124]. There has been little research on the direct conversion of Th1 cells to Treg cells. Kanamori et al. established a method for Treg inducing by resting Th1 cells without T-cell receptor ligation before stimulation in the presence of TGF-β. They found that Foxp3 expression was promoted with reduced mammalian target of rapamycin complex 1 (mTORC1) activity in Th1 cells [125]. The conversion between Th2 and Treg cells is a complex process that is influenced by various factors including genetic and epigenetic programming. Memory Th2 cells were able to shift to Foxp3^+^ T cells by TGF-β when stimulated in the presence of *all-trans* retinoic acid and rapamycin [126]. In contrast, IL-4 was found able to promote the development of Th2 cells from Treg cells [127]. In the absence of signal transduction activator (Pak2) expression in Treg cells, these cells can express the transcription factor GATA3 along with Th2-related cytokines, which shifts Treg cells into Th2 cells [128]. It was found that IL-6 can prompt the conversion of nTreg cells into Th17 cells in a TGF-β-dependent manner. IL-6-induced IL-17-producing cells can self-induce even without exogenous TGF-β [129]. However, Zheng et al. found that Treg cells induced by IL-2 and TGF-β are resistant to Th17 transformation. For IL-2 and TGF-β down-regulated IL-6 receptor expression in the induced Treg cells [130]. Treg cells were found to convert into functional Tfh cells in Peyer’s patches and in atherosclerosis, which correlates with increased IL-6α expression as well as decreased IL-2Rα and phosphorylated STAT-5 expression [131–133]. IL-2 can convert Tfh cells to follicular Treg cells in patients with systemic lupus erythematosus [134]. Indicating that Treg cells and other CD4^+^ T cell subsets can convert to each other, however, it is a complex process that is influenced by various factors, including the cytokine environment and the expression of specific transcription factors. Further research is needed to fully understand the mechanisms that regulate this conversion and its implications for immune function and disease.

### Costimulatory molecules on Foxp3^+^ Treg cells

**Figure 3:**
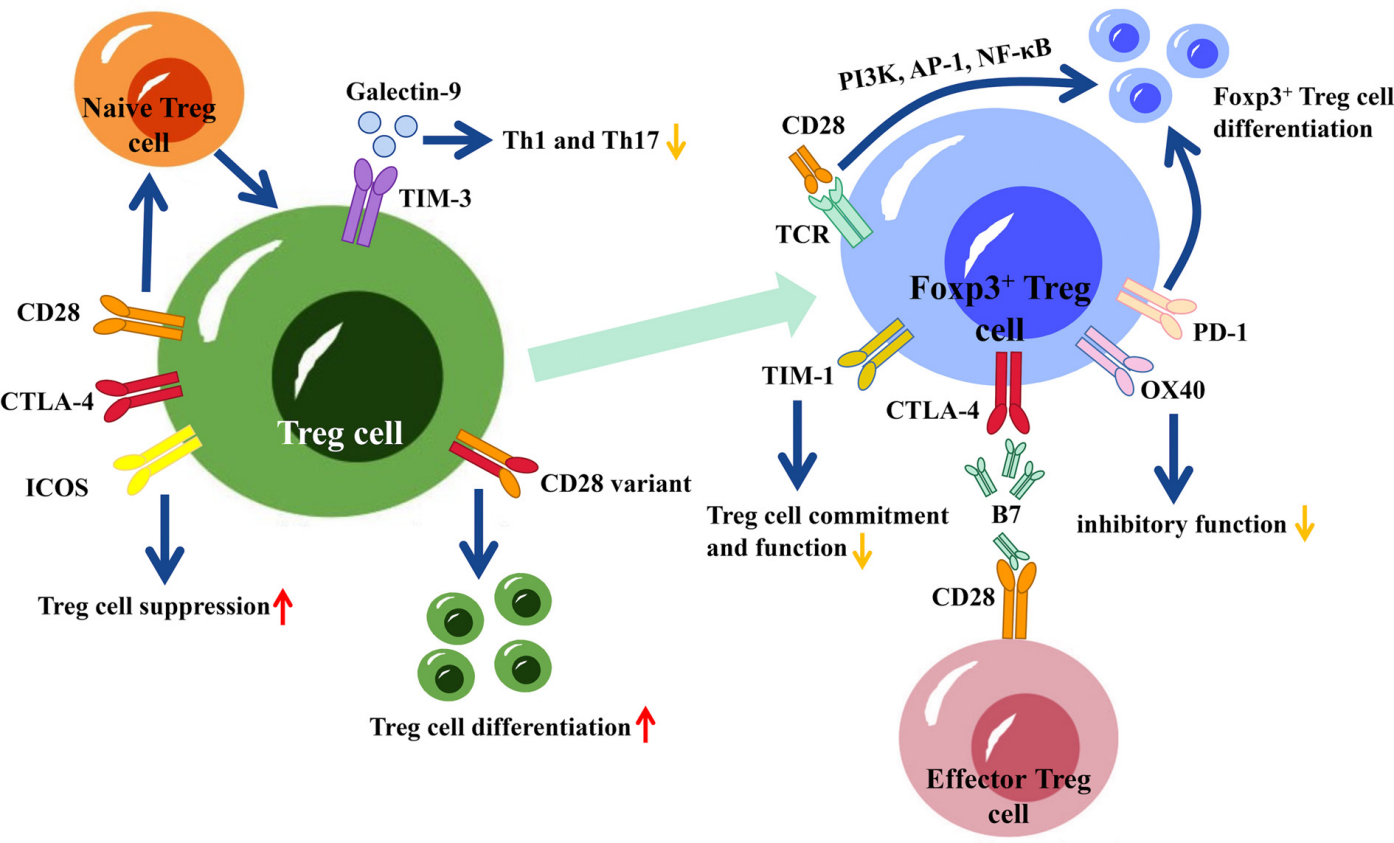
Co-stimulatory molecules and their effects on forkhead box P3 (Foxp3)^+^ regulatory T (Treg) cells CD28, Cytotoxic T-lymphocyte-associated protein 4 (CTLA-4), and inducible costimulator (ICOS) are involved in Treg cell activation. ICOS has the potential to enhance the suppressive capabilities of Treg cells, while CD28 plays a role in facilitating the conversion of naïve Treg cells into mature Treg cells. Additionally, a variant of CD28 can trigger the differentiation of Treg cells. T-cell immunoglobulin and mucin domain (TIM)-3 is consistently present on natural Treg cells, and it engages with Galectin-9 to diminish the secretion of type 1 T helper (Th1) and Th17 cytokines while increasing the proportion of Treg cells. CD28 and T-cell receptor (TCR) co-stimulate the differentiation of Foxp3^+^ Treg cells by activating phosphoinositide 3-kinases (PI3K), activator protein 1 (AP-1) and nuclear factor kappa-B (NF-κB) signaling pathways. CTLA-4 is more predominantly present in Foxp3^+^ Treg cells, and the affinity of the CTLA-4 receptor on Foxp3^+^ Treg cells is higher than that of CD28, enabling Treg cells to bind more easily to cytokines such as B7 in the physiological environment. Programmed cell death 1 (PD-1) is able to assist in the differentiation and maintenance of Foxp3^+^ Treg cells. OX40 signaling inhibits the TGF-β-mediated induction of Treg cells. T-cell immunoglobulin and mucin domain (TIM)-1 is involved in the differentiation of Th1, Th2, and Th17 cells while inhibiting both the commitment to and suppressive function of the Treg cell phenotype.

Costimulatory molecules play an active role in co-signaling with the initial activating signals, contributing to T-cell differentiation and fate. They are critical for the complex control of Treg cell development and function [135,136]. There are several costimulatory molecules including CD28, CTLA-4, ICOS, programmed cell death 1 (PD-1), T-cell immunoglobulin and mucin domain (TIM)-1, TIM-3, glucocorticoid-induced tumor necrosis factor receptor (GITR) and OX40 expressing on Treg cells (Figure 3).

The immunoglobulin-related receptor family, which includes CD28, CTLA-4, ICOS and other proteins, can promote Treg cell activation [69,137]. CD28 is critical to the development of naïve Treg cells in the thymus [136]. CD28 and TCR costimulate the differentiation of Foxp3^+^ Treg cells by activating PI3K, AP-1, NF-κB and other related signaling pathways [137,138]. Foxp3^+^ Treg cells express CTLA-4, which is homologous to the CD28 on effector T cells, resulting in a competitive relationship between the two [69]. Compared with CD28, CTLA-4 is more predominantly present in Foxp3+Treg cells, and the affinity of the CTLA-4 receptor on Foxp3^+^ Treg cells is higher than that of CD28, enabling Treg cells to bind more susceptible to cytokines such as B7 in the physiological environment [69,70,137]. However, CTLA-4 can dimerize with CD28, resulting in the formation of a high-affinity CD28 variant that can participate in positive co-stimulation, leading to the activation of Treg cell differentiation [69,139]. The costimulatory molecule ICOS plays a role in promoting IL-2-induced cell survival and proliferation, enhancing the inhibitory activity of Treg cells [140].

In addition to CTLA-4, PD-1 also plays a central role in both the differentiation and maintenance of Foxp3^+^ Treg cells. This results in an increased generation of Treg cells and enhances their suppressor function with sustained Foxp3 expression [141]. However, PD-1-mediated inhibitory effects were demonstrated independently of CTLA-4 [142]. Additionally, specific blockade of the PDL-1-B7-1 pathway leads to a reduction in the Treg population, indicating its involvement in Treg homeostasis. TIM-3 is constitutively expressed on natural Treg cells and can interact with Galectin-9 for Treg function [143]. Blocking TIM-3 reduces the suppressive ability of natural Treg cells and stops the formation of allospecific adaptive Treg cells, preventing or cancelling induced peripheral tolerance [144,145]. Therefore, administering soluble Galectin-9 will decrease the production of Th1 and Th17 cytokines and increase the proportion of Treg cells, which will further promote allograft survival [136,146]. GITR is considered a promising therapeutic target in cancer and autoimmune disease, for it can activate signaling pathways. GITR is also thought to be involved in the regulation of Foxp3^+^ Treg cells due to its constitutive expression on Treg cells. However, the precise role of GITR is still unknown, especially in transplantation [136].

OX40 and TIM-1 signaling negatively affect Treg cells. OX40 signaling can inhibit the TGF-β-mediated induction of Foxp3^+^ Treg cells from both naïve CD4^+^ T cells and CD4^+^ T effector cells [147,148]. OX40 ligation will result in reduced Foxp3 expression, while OX40 stimulation causes the abrogation of natural Foxp3^+^ Treg cell suppressive function [149]. Nevertheless, OX40 is not implicated in the homeostatic regulation of Treg cells [148]. TIM-1 is involved in the differentiation of Th1, Th2 and Th17 cells while inhibiting both the commitment to and suppressive function of the Treg cell phenotype [150,151].

## The role of Treg cells in human lung diseases

### Asthma

Asthma is a chronic heterogeneous disease and is characterized by heightened inflammation and mediator release caused by overactive Th2 cells [152]. The pathogenesis of asthma is marked by an abnormal Th cell immune response, where Treg cells have impaired immunosuppressive function, while effector Th cells are overreactive [153]. It was found that Treg/Th17 cell imbalance is a promoter of asthma development [154]. In human allergic asthma, there is an inflammatory environment characterized by high levels of the chemokine C-C motif chemokine ligand (CCL)20, which can hinder the TGF-β-induced differentiation of Treg cells while facilitating the migration of CCR6^+^ Treg cells. Treg cells expressing CCR6 have the potential to promote inflammation and exacerbate allergic asthma. Therefore, inhibiting the CCR6-CCL20 pathway may be advantageous in preventing the transformation of Treg cells specific to house dust mites into Th17-like cells. [155,156].

In contrast with Th cells, Treg cells are major contributors to the regulation of asthma by negatively modulating Th1, Th2 and Th17 cells, which helps to prevent the overactivity of these cells [157]. Treg cells prevent airway inflammation by producing the anti-inflammatory cytokines IL-10 and TGF-β, which can inhibit the proliferation of ILC2s and granulocytes, thereby regulating autoimmune effects [158,159]. The suppression of Th2 inflammation by Treg cells is achieved through the action of the transcription factor BCL6, which hinders the expression of GATA3, a transcription factor necessary for the production of IL-4 and IL-13. Under inflammatory conditions, Treg cells promote their stability by expressing BCL6 [153,159]. Ding et al. found that down-regulation of the expression of glucocorticoid-induced tumour necrosis factor receptor ligand (GITRL) on the surface of DCs will induce tolerogenic Treg cell skewing in asthma, which further facilitated the apoptosis of pathogenic Th2 and Th17 cells [157]. Yao et al. found that the suppressive function and stability of Treg cells are enhanced by androgen receptor signaling, which down-regulates ST2 expression on Treg cells and reduces allergen-induced IL-33 expression in airway epithelial cells. In addition, Treg cells were demonstrated to improve airway remodeling and restore Th1/Th2 balance through the DLL4-Notch signaling pathway [160,161].

In the context of catabolism, there exists an enzyme known as heme oxygenase 1 (HO-1) with anti-inflammatory properties. Increasing HO-1 transcription has the capacity to enhance the expression of Foxp3 and IL-10 within Treg cells, along with an increase in the transcription of TGF-β1. Additionally, HO-1 can induce the up-regulation of Foxp3 and anti-inflammatory cytokines in cells that did not previously express them. This mechanism serves as a protective response against airway inflammation in asthma and is mediated by Treg cells, IL-10, and membrane-bound TGF-β1 [162]. Furthermore, there is the discovery of a novel interleukin cytokine, IL-38, which shares functional similarities with IL-36Ra [163]. While IL-38 can be secreted by various cell types, no existing literature confirms its production by Treg cells. It appears to be a widespread anti-inflammatory factor. Notably, in the presence of elevated periostin concentrations, IL-38 is inversely correlated with the percentage of CD4^+^CD25^high^Foxp3^+^ Treg and CD4^+^CD25^+^ T cells. This suggests a potential connection between IL-38 and suppressive Treg cells in the context of asthma development. However, further research is needed to elucidate the mechanism through which these two factors collaborate to inhibit bronchial inflammation in asthma [164,165].

### Acute respiratory distress syndrome (ARDS)

ARDS is a pulmonary inflammatory disorder characterized by cytokine-induced inflammation [166]. ARDS development is linked to an imbalance between pro- and anti-inflammatory factors in the lungs, and an elevated Th17/Treg ratio is a risk factor for early ARDS onset [167,168]. It was found that secretory phosphoprotein 1 (SPP1) suppresses VHL expression, thereby decreasing the ubiquitination and degradation of hypoxia-inducible factor 1α (HIF-1α), resulting in an increase in the Th17/Treg ratio and exacerbation of inflammation in ARDS [167,169].

Luteolin, which is derived from certain fruits and vegetables such as apples and broccoli, has a preventive effect by reducing lung inflammation and injury. It was found that luteolin can activate Treg cells to reduce the symptoms of ARDS [170]. When induced by luteolin, there is accelerated differentiation of CD4^+^CD25^+^ T cells into CD4^+^CD25^+^Foxp3^+^ Treg cells in BALF and serum, which is accompanied by an increase in IL-10 production [171]. Treg cells have been found to alleviate caspase-11-mediated inflammatory cell death in lung tissue [170]. Curcumin is the active component found in the herb turmeric, which has been found to have anti-inflammatory properties that enhance the ability of Treg cells to combat ARDS [172]. Similar to luteolin, curcumin increases the rate of differentiation of initial CD4^+^ T cells into CD4^+^CD25^+^Foxp3^+^ Treg cells.

ARDS is a severe form of acute lung injury (ALI) [173]. As evidenced by their accumulation in the BALF of ALI patients, Treg cells promote inflammation regression in ALI patients by inducing cytokine TGF-β1 and neutrophil apoptosis. Furthermore, Treg cells can modulate fibrocyte recruitment to the lung in LPS-induced injury, which reduces fibroplasia in ALI patients [168]. In a mouse model of ALI, Treg cells suppressed fibroblast recruitment along the CXCL12/CXCR4 axis, thereby reducing fibrotic proliferation [174]. Ge Gen Scutellaria Tang (GQD), an herbal remedy for inflammatory lung diseases, has been shown to improve the degree of lung damage in ALI. GQD stimulates Treg cell activity and promotes the expression of CYP1A1 and cytokine TGF-β while reducing the level of Th17 cells. Such Treg cell stimulation further restores the Treg/Th17 balance, thus reducing the degree of pulmonary edema [175].

### Idiopathic pulmonary fibrosis (IPF)

IPF is a chronic and progressive pulmonary disease characterized by the gradual scarring of lung tissue, leading to a decline in lung function [174,176,177]. In IPF patients, Treg cells fail to suppress the secretion of Th1 and Th2 cytokines, resulting in a deficiency of Treg cells [178]. The presence of PD-1 on the surface of CD4^+^ T cells, along with the production of TGF-β1 and IL-17A, can inhibit Treg cell transformation and promote IPF development [179]. The development of IPF is exacerbated by a significant increase in the number of activated Treg cells in the peripheral blood of patients. Likewise, quiescent Treg cells generated in the thymus can undergo proliferation and differentiate into activated Treg cells, which also exhibit immunosuppressive properties and enhance the population of cytokine-secreting CD45RA^−^CD25^+^ cells [180].

Treg cells have a dual role in pulmonary fibrosis depending on the disease stage. In the early stage, they contribute to the development of fibrosis by promoting TGF-β1 release, reducing the Th17 response, stimulating the aggregation of fibroblasts, increasing the deposition of collagen and participating in the progression of pulmonary fibrosis [181]. Conversely, in the late stage, Treg cells have a protective function by suppressing the production of CXCL10 and fibroblasts, thereby decreasing fibroblast recruitment and mitigating the extent of pulmonary fibrosis [174,181]. Treg cells also mediate the transfer of proteins FGF9 and CCL2 to ameliorate bleomycin-induced pulmonary fibrosis [177].

### Chronic obstructive pulmonary disease (COPD)

COPD is a severe inflammatory disease characterized by heightened pro-inflammatory cytokine levels and increased reactive oxygen species in the peripheral blood [182]. An imbalance in the ratio of Th17 to Treg cells plays a significant role in the initiation and development of the inflammatory response in COPD [156]. This imbalance results in a decrease in the expression of intracellular Foxp3 mRNA, which in turn affects the secretion of anti-inflammatory factors like IL-10 and TGF-β by Treg cells. In the absence of adequate IL-10 levels, IL-23 levels increase, thereby accelerating the differentiation of Th17 cells. Th17 cells, which express RORγt, release the pro-inflammatory cytokines IL-17A, IL-17F and IL-22 [183]. These cytokines induce the production of neutrophil growth factor GM-CSF, chemokines CXCL1 and CXCL8, and antimicrobial peptides by epithelial cells to promote neutrophil accumulation in the airway [184].

In clinical practice, the administration of oral N-acetylcysteine (NAC) can reduce cytokine activity and prevent neutrophil aggregation, which reduces inflammation, in the lungs. These effects are evident through increased levels of IL-10 in COPD patients, modulation of Th1/Th2 cell balance, and increased differentiation of CD4^+^ T cells into Treg cells [182]. In COPD patients, the inflammatory cytokine IL-17 is responsible for directing neutrophil aggregation to the airways [183]. This effect is synergized by HIF-1α and RORγt protein expression. While NAC treatment can help alleviate the inflammatory environment of COPD by reducing HIF-1α expression [13,34]. Xuanbai Chengqi decoction (XBCQ) is an herbal medicine used to treat COPD patients in the exacerbation stage. Moderate doses of XBCQ can restore Treg/Th17 balance, suppress Treg cell deficiency in the lung, reduce the expression of pro-inflammatory factors such as IL-1β and TNF-α, inhibit inflammatory infiltration, and significantly improve lung injury caused by COPD [184]. Furthermore, administration of the anticholinergic drug tiotropium bromide and the long-acting β2 agonist olodaterol to PBMCs from COPD patients has been shown to elevate the proportion of Foxp3^+^ Treg cells and reduce the proportion of T cells that express both acetylcholine IL-17A and acetylcholine RORγt in PBMCs. This effectively controls the development of inflammation in COPD [169].

### Cystic fibrosis (CF)

CF is an autosomal recessive genetic disorder resulting from mutations in the cystic fibrosis transmembrane conductance regulator (CFTR) gene [185]. The hallmark of CF is chronic unremitting lung disease, a major cause of morbidity and mortality in CF patients. This condition is characterized by chronic infections, immune dysfunction, and dysregulated airway inflammatory responses, evidenced by decreased Treg cell activity and excessive inflammatory reactions [186,187]. Treg cells, especially CD4^+^CD25^+^ Treg cells, play a crucial role in maintaining the balance between anti-inflammatory and pro-inflammatory mediators, thereby stabilizing the immune system [187,188]. These cells help prevent excessive inflammation by inhibiting the hyperactivity of certain T cell subsets, such as Th17 or Th2 cells [189]. However, in CF lung disease, there is an elevation in pro-inflammatory cytokines such as IL-6, IL-8, tumor necrosis factor-α and IL-1β, along with a decrease in anti-inflammatory cytokine IL-10 and anti-inflammatory mediator lipoxin levels [190]. This dysregulation of Th17/Th2 cells contributes to the development of chronic lung disease in CF [189]. Moreover, the deficiency in the number or function of Treg cells may also contribute to CF lung disease. Studies have shown that compared with healthy controls, patients with CF lung disease have significantly lower Treg cell counts in their peripheral blood, with a reduced proportion of Treg cells in CD4^+^ cells [190]. In the context of *Pseudomonas aeruginosa* infection, CFTR and *Pseudomonas aeruginosa* act as major mediators of Treg cell functional impairment in patients with CF lung disease [188]. Interestingly, Treg cells in patients with Type 1 diabetes are dysfunctional, leading to the secretion of the pro-inflammatory factor IL-17 [191].

The positive correlation between the number of Treg cells and lung function in CF patients suggests that restoring T-cell responses can be achieved by increasing Treg cell numbers and/or enhancing their function. One approach is by providing chemically modified Foxp3 mRNA or enhancing indoleamine 2,3-dioxygenase (IDO) activity, as demonstrated in a recent study showing the association between IDO dysfunction and Treg cell dysregulation in Cftr^−/−^ mice [188]. This presents a promising treatment strategy for CF lung disease. Another potential therapeutic approach involves direct modulation of T-cell responses to counteract the excessive inflammatory response observed in CF [192]. As CF is a systemic disease that affects the absorption of fat-soluble vitamins A and D, treating CF patients with 1,25(OH)2-vitamin D3 has shown positive effects. This treatment leads to increased IL-10 levels in Treg cells, resulting in elevated expression of TGF-β1 transcripts and proportion of TGF-β on CD4^+^CD25^+^ cells, and decreased Th2 responses in CD4^+^ T cells [193]. Furthermore, highly effective CFTR modulators have been developed to improve the quality of life for patients with multiple CFTR mutations. Treatment with Elexacaftor/tezacaftor/ivacaftor (ELX/TEZ/IVA) has been shown to down-regulate blood levels of pro-inflammatory factors IL-6, IL-8 and IL-17A and up-regulate the percentages of Treg cells in CF patients [186]. ELX/TEZ/IVA, the first approved triple CFTR modulator therapy, effectively addresses inflammation and immune dysregulation caused by CFTR deficiency [194]. These findings provide hope for more effective and targeted therapies in managing CF lung disease.

### Lung cancer

Based on their histological features, lung cancer can be categorized into two histological types: NSCLC and small cell lung cancer (SCLC). NSCLC is the most prevalent form, constituting approximately 80% of lung cancer cases. NSCLC further subdivides into adenocarcinoma, large cell carcinoma and squamous cell carcinoma [195,196]. While Treg cells play a role in suppressing inflammation in immune responses, they also possess dual functions of aiding cancer cells in evading immune surveillance in cancer immunity and promoting both tissue repair and immune suppression [197]. Treg cells were found to hinder effective anti-tumor immunity in patients with tumors, and contribute to the development of diverse malignancies, including NSCLC [198].

In typical physiological conditions, effector T cells engage with activated APCs, presenting processed antigenic peptides through MHC molecules. Subsequently, neighboring T cells interact with the antigen/MHC complex via the TCR, leading to additional stimulatory signals, including the involvement of CD28 proteins on T cells in conjunction with B7 proteins found on the surface of APCs. This heightened interaction enhances T-cell activation and promotes an effective immune response [199]. However, as the immune response wanes, the expression of molecules such as CTLA-4 and PD-1 on the surface of CD8^+^ T cells increases. These cooperative inhibitory molecules interact with ligands in the tumor microenvironment (TME), ultimately inducing T-cell exhaustion [196]. Treg cells that express TIM-3 exhibit heightened activation and greater immunosuppressive activity compared with TIM-3-negative Treg cells. Consequently, the co-expression of TIM-3 and PD-1 on Treg cells serves as a marker for the depletion of CD8^+^ T cells in human tumors [200].

Treg cells employ various mechanisms to exert their role of maintaining immune self-tolerance and suppressing the activation of effector T cells. One such mechanism is Treg cells homing into tumour tissues, guided by the gradient of CCR4 ligands such as CCL17 and CCL22. Once there, they release immunosuppressive cytokines such as TGF-β and IL-10, which serve to facilitate immune evasion by tumors through the inhibition of effector T-cell activation and function [201,202]. Another important mechanism involves the interaction of CTLA-4 produced by Treg cells with the B7 protein on the surface of APCs. This interaction can impair the immune system’s ability to mount an effective anti-tumor response. CTLA-4 expressed by effector Treg cells binds to CD80 or CD86 molecules on APCs with higher affinity than CD28 molecules, resulting in a more potent inhibitory signal to APCs and rendering effector T cells unresponsive. This interaction can be blocked by anti-CTLA-4 antibodies, thereby restoring the anti-tumor immune function of immune cells [17,199,203]. Third, Treg cells can directly inhibit the anti-tumor activity of immune cells by interacting with NK cells and DCs. The high-affinity receptor CD25 on the surface of effector Treg cells enables them to deprive IL-2, inhibiting the activation and tumor-clearing function of NK cells and CD8^+^ T cells [204]. In the context of lymph nodes draining from lung tumours, Treg cells prevent the activation of T cells and their recognition of tumor antigen proteins presented by DCs. Treg cells achieve this by hindering the expression of stimulatory proteins on the surface of DCs [205,206]. Within mediastinal lymph nodes, this inhibition occurs in a spatially coordinated manner and relies on MHC II interactions between Treg cells and type 1 DCs [207]. Furthermore, Treg cells within tumor-associated tertiary lymphoid structures (TA-TLS), acting as potent T-cell suppressors, often express high levels of the enzyme CD39. Together with CD73, this enzyme generates adenosine, which further promotes tumour growth and immune evasion [208,209].

The TME plays a pivotal role in the initiation and progression of tumors, and the development of tumors relies on an appropriate TME. Interactions between tumor cells and the surrounding stroma within the TME are a significant source of intratumoural heterogeneity [210,211]. In NSCLC, the TME of patients with lymph node metastasis or advanced disease stages exhibits elevated production of TGF-β. This promotes the infiltration of effector Treg cells into the TME and increases the expression of inhibitory molecules such as GITR and PD-L1 on the surface of APCs, thereby enhancing the immunosuppressive function of Treg cells. Moreover, within the TME, Treg cells are recruited by CCL20 produced by tumor cells, which bind to the receptor CCR6. Additionally, heightened glycolytic activity in cancer cells provides ample fatty acids and lactic acid, fueling the proliferation of Treg cells [17,212,213]. In the mediastinal lymph nodes of cancer patients, increased levels of IFN-γ promote the transformation of Treg cells into Th1-like effector Treg cells. This transformation increases their interaction with type 1DCs, ultimately inhibiting the activity of CD8^+^ T cells. The chemokine CXCL13 is predominantly expressed in advanced CD8^+^ T cells and Treg cells. When co-expressed with its receptor CXCR5, it can enhance tumor evasion and invasion [210,214]. It was found that vascular endothelial growth factor (VEGF), a growth factor involved in angiogenesis, contributes to immunosuppression within the TME. VEGF-A exerts its immunosuppressive effect by increasing the number of immature dendritic cells, promoting Treg cell recruitment through VEGFR-2 binding, and enhancing Treg cell infiltration in the TME by binding to NRP-1 [198]. Additionally, FOXC1, a transcription factor, promotes the expression of LINC00301 in NSCLC. LINC00301 promotes TGF-β1 secretion in lung tumors to attract Treg cells, which will further result in immunosuppression via facilitating the inhibition of CD8^+^ T cell differentiation and HIF-1α up-regulation [215].

Lung cancer immunotherapy presents significant challenges, with tumor cells having the ability to evade immune surveillance and dampen overall immune responses through the secretion of immunosuppressive cytokines by Treg cells and reduced expression of MHC molecules [216]. However, there are potential avenues for intervention in lung cancer treatment. Anti-CCR4 antibodies can be employed to hinder the recruitment of Treg cells, anti-CD25 antibodies like docetaxel and cyclophosphamide can be used to reduce the number of Treg cells, and anti-TGF-β drugs can be employed to counteract the immunosuppressive effects of cytokines secreted by Treg cells within the TME [199,217,218]. Dasatinib, an antitumor medication, has been shown in clinical settings to inhibit the differentiation and function of CD4^+^CD25^+^ Treg cells in peripheral blood, leading to a reduction in the inhibition of CD8^+^ T cells [219]. Epacadostat, an inhibitor of the immunomodulatory molecule indoleamine 2,3-dioxygenase-1, when combined with dasatinib in the treatment of NSCLC, reduces Treg cells in the lung TME and reactivates immunomodulatory mechanisms while suppressing tumor immunosuppression caused by Treg cells [220]. The administration of anti-PD-1 antibodies boosts effector T-cell activation within tumors and prevents Treg cell differentiation. In NSCLC, the combination of anti-PD-1 and dasatinib has been discovered to completely inhibit the proliferation and differentiation of tumor-infiltrating Treg cells, reduce Treg cell levels, induce tumor regression, and elicit immune memory [221].

### Swelling and inflammation in the main passages (bronchial tubes) that carry air to the lungs (bronchitis)

It has been discovered that acute bronchitis, an inflammation of the bronchi in the lungs, is associated with an imbalance in the Th17/Treg ratio in children. This imbalance is indicated by an increase in serum levels of IL-17 and IL-22 cytokines, as well as a decrease in serum levels of IL-10 and TGF-β. This suggests that the Th17/Treg ratio plays a role in the development of acute bronchitis [222,223]. Dysfunctional Treg cells are a significant factor in severe viral bronchiolitis in infants, as defective Treg cells produce lower levels of CD39 compared with healthy Treg cells, and healthy Treg cells are capable of blocking allergen-induced HMGB1 translocation [224]. Respiratory syncytial virus (RSV), a common respiratory virus, is a leading cause of bronchitis and pneumonia in infants and the elderly [225,226]. Infants infected with RSV show decreased levels of activated Treg cells, as well as significantly reduced levels of alarmin IL-33 and increased levels of Th1, Th2, and pro-inflammatory cytokines [227]. Studies have indicated that following RSV infection, there is an excessive secretion of IL-8, resulting in increased levels of Th17-related factors, including IL-17, IL-23 and RORγt. However, there is minimal alteration in the Treg subpopulation, and the levels of IL-10, TGF-β, and FOXP3 decrease. This shift in the Th17/Treg ratio contributes to symptoms like bronchial inflammation, collapse and deformation, underscoring the impact of Treg cell numbers on the development of bronchial inflammation [228–231]. These findings highlight the significance of Treg cell quantity in the context of bronchial swelling and inflammation.

Furthermore, the research findings suggest that bronchiolitis obliterans syndrome (BOS) is linked to a reduction in Treg cell levels. These Treg cells employ a non-contact immunosuppressive mechanism by secreting immunosuppressive cytokines like TGF-β and IL-10, thereby promoting tolerance and resolving inflammation [232,233]. Additionally, BOS patients exhibit increased senescent CD28^null^ T cells and natural killer T-like cells in their peripheral blood [234]. TGF-β exerts its inhibitory effects by suppressing the cytotoxic activity of NK cells and the expression of IFN-γ and T-bet. It also hinders macrophage phagocytic function by down-regulating receptors responsible for the phagocytosis of bacteria, senescent cells, and apoptotic cells. Furthermore, TGF-β dampens antigen presentation by macrophages, reducing the expression of MHC class II molecules and inflammatory cytokines, thereby achieving immunosuppression. Treg cells contribute to immune suppression through both direct cell-to-cell contact and the production of cytokines like galectin-1 and IL-35 [235,236]. They also inhibit the activation and functional roles of T cells generated in antigen-specific immune responses, thereby fostering immune tolerance to self-antigens and suppressing autoimmune reactions.

### Lung infection (pneumonia)

Pneumonia stands as a significant global health concern, being one of the leading causes of morbidity and mortality worldwide and representing the top infectious cause of death [237]. It is an inflammatory lung disease primarily caused by pathogens, leading to the release of excessive inflammatory cytokines and rendering individuals susceptible to secondary infections for an extended period [238]. The development of pneumonia can disrupt the balance between Th17 cells and Treg cells, leading to acute inflammatory damage in the lungs, potentially progressing into severe conditions like ARDS or sepsis [239].

Treg cells can critically function as immunosuppressors in infectious diseases by producing abundant anti-inflammatory cytokines like TGF-β, IL-10 and IL-35 [240]. Additionally, Foxp3^+^CD39^+^ Treg cells can help eliminate inflammasome-mediated pro-inflammatory responses by hydrolyzing ATP [241]. Treg cells also contribute to the reduction of lung inflammation and promote lung repair by interacting with macrophages and alveolar epithelial cells [242]. Moreover, in healthy tissues, Treg cells are essential for maintaining immune homeostasis and are capable of assembling rapidly in response to viral attacks in the lungs to aid in tissue repair. They actively promote tissue regeneration following injury by producing repair mediators like EGF receptor ligand-deregulated proteins [243]. Overall, the multifaceted functions of Treg cells serve to regulate an overactive immune system and support the maintenance and repair of healthy tissues in various contexts.

Mycoplasma pneumoniae (MP) is a common extracellular pathogen responsible for up to 40% of community-acquired pneumonia (CAP) cases in children. MP attaches to ciliated epithelial cells of the respiratory mucosa, activating various signaling pathways and causing lung damage [244,245]. The severity of lung injury in patients with MP pneumonitis is associated with an imbalance between Treg and Th17 cells [246]. In MP pneumonia patients, Treg cells exert anti-inflammatory effects by inhibiting immune cells, such as CD4+ and CD8+ T cells, B cells, dendritic cells, and NK cells, through contact-dependent inhibition and the release of anti-inflammatory cytokines IL-10 and TGF-β1 [245,247].

Pseudomonas aeruginosa (PA) is known to cause chronic and acute lung infections in immunodeficient or immunosuppressed patients, leading to inflammation, elevated levels of pro-inflammatory cytokines and chemokines, and substantial neutrophil recruitment [240,248]. In mouse lung tissue, PA infection can result in increased inflammation, up-regulation of pro-inflammatory cytokines such as TNF-α, IL-17 and IL-6, and an amplified Th17 cell response, despite CAP being an infrequent outcome of PA infection [237,249]. However, the involvement of Treg cells in the host response against CAP has not been directly proven [250].

Respiratory syncytial virus (RSV) is a significant cause of viral pneumonia and bronchiolitis in children worldwide. RSV’s ability to evade normal immune memory underscores the critical role of effective T-cell responses in clearing RSV infections [251]. During RSV infection of the lungs, Foxp3^+^ Treg cells exert a critical role in maintaining immune homeostasis and can suppress inflammation in healthy individuals. In response to viral infection of the respiratory tract, Treg cells in the lung produce the anti-inflammatory cytokine IL-10 through the transcriptional regulator B lymphocyte-inducible maturation protein 1 (BLIMP1), thus regulating excessive immune responses [252]. Studies investigating respiratory syncytial virus models have shown that the depletion of Treg cells can lead to delayed migration of CD8^+^ T cell subpopulations [253]. Similarly, in studies using Influenza A virus models in mice, infected individuals exhibit a marked induction of CD4^+^Foxp3^+^ T cells, but no significant effects on mortality, viral clearance, or lung tissue cellularity have been demonstrated [253,254].

Coronavirus disease 2019 (COVID-19), caused by the novel coronavirus Severe Acute Respiratory Syndrome Coronavirus 2, is characterized by a crucial role of cytokine storms in its pathogenesis. An excessive immune response fails to clear the virus but instead exacerbates respiratory distress and damages the heart and other organs [255–257]. Normally, Treg cells act as the first line of defense against viral infections and uncontrolled inflammation, inhibiting CD4^+^ and CD8^+^ T cell responses and reducing infiltration of neutrophils, NK cells and eosinophils [258]. However, severe COVID-19 patients exhibit increased levels of cytokines like IL-1β, IL-6, IL-7, IL-10 and IL-17, as well as the chemokine IL-8, accompanied by deficiencies in the regulation of pro-inflammatory responses by Treg cells due to reduced Treg cell numbers and decreased Treg/Th17 cell ratios [259,260]. It was demonstrated that the inhibition of Treg cell differentiation by STAT-3-mediated signaling further exacerbates cytokine storms and promotes immunopathological responses [261]. Therefore, Treg cells act as crucial modulators of immune responses during pneumonia, ensuring an appropriate balance between inflammation and resolution, ultimately supporting effective defense and recovery.

### Blocked lung artery (pulmonary embolus)

Deep vein thrombosis refers to the condition in which blood clots develop in the deep veins of the body [262]. This condition may lead to serious complications such as pulmonary embolism, post-thrombotic syndrome, and chronic thromboembolic pulmonary hypertension [263]. Treg cells have been found to accumulate in thrombosed veins, where they form the matricellular acid- and cysteine-rich protein SPARC (secreted protein acidic and rich in cysteine) within the clot, regulate matrix metalloproteinase activity, recruit monocytes and respond to TGF-β cytokine to facilitate clot regression [264]. The study has delved into the phenotype of Treg cells within thrombi and the walls of thrombosed veins, revealing that Treg cells in the vicinity became activated upon recruitment. This activation was evident through the significant up-regulation of CD69 expression in Treg cells, with levels steadily increasing as the thrombus aged. The specific mechanisms of action of Treg cells in pulmonary embolism have not yet been thoroughly studied, and research in this area is ongoing. However, Treg cells are generally known for their immunosuppressive and anti-inflammatory functions, which might potentially help prevent excessive inflammation and immune-mediated damage to lung tissue through immune modulation. Treg cells are potentially active in preventing excessive inflammation and immune-related damage to lung tissue. They achieve this regulation of immunity by producing cytokines like IL-10 and TGF-β to dampen the inflammatory response, thereby potentially reducing inflammation linked with pulmonary embolism [265,266]. Furthermore, Treg cells may contribute to tissue repair and regeneration [267,268] and could play a role in repairing damaged lung tissue in cases of pulmonary embolism. Additionally, Treg cells have the capacity to curtail excessive immune activation [19,269]. In the pulmonary vascular system, pulmonary embolism might trigger an immune response due to the presence of a thrombus or embolus. Treg cells can help counteract an overload of the immune response brought about by pulmonary embolism. It’s crucial to emphasize that the precise mechanisms through which Treg cells function in pulmonary embolism may differ among individuals and according to the unique circumstances of the disease. Additional research is required to gain a comprehensive understanding of Treg cell involvement in pulmonary embolism and to explore their potential application in therapeutic strategies.

### The role of Treg cells following lung transplantation

Although lung transplantation is currently the only treatment for end-stage lung disease [270], the survival rate after lung transplantation is significantly lower compared with that of other organs. This is due to the high immunogenicity of the lung and continuous exposure to the external environment [271]. In the early 1980s, the technical obstacles of lung transplantation were overcome. However, the main challenge in achieving survival in lung transplant patients is chronic rejection known as bronchiolitis obliterans (BO) [272]. Approximately 50% of the patients who survive more than three months post-transplantation are expected to develop this complication. BO is a chronic rejection response that can occur one to several years after lung transplantation, leading to progressive fibrosis of the small airways and a gradual reduction in lung ventilation [273]. Following lung transplantation, chronic rejection occurs at the location where type V collagen (COLV) is exposed [274]. COLV is a small fibrous collagen that is expressed by small airway epithelial cells and is located in the peribronchial and perivascular tissues of the lung [275]. After lung transplantation, matrix metalloproteinases can cleave collagen, which exposes COLV and releases its antigen fragment. This results in increased levels of COLV in patients with BO after transplantation. This antigen fragment plays a role in the development of BO. In addition to COLV, K-α1-tubulin also contributes to autoimmune-mediated BO development and chronic rejection [276]. IL-17 is associated with the development of post-transplant BO, and the autoantibodies to COLV and K-α1 tubulin in lung transplant recipients are IL-17 dependent. In mouse studies, the inhibition of IL-17 resulted in decreased levels of autoantibodies to COLV and K-α1 tubulin [276,277]. In addition, there was a significant increase in the level of IL-17 in the bronchial lavage fluid of lung transplant patients, suggesting that IL-17 may have a key role in the development of BO following lung transplantation [278].

**Figure 4:**
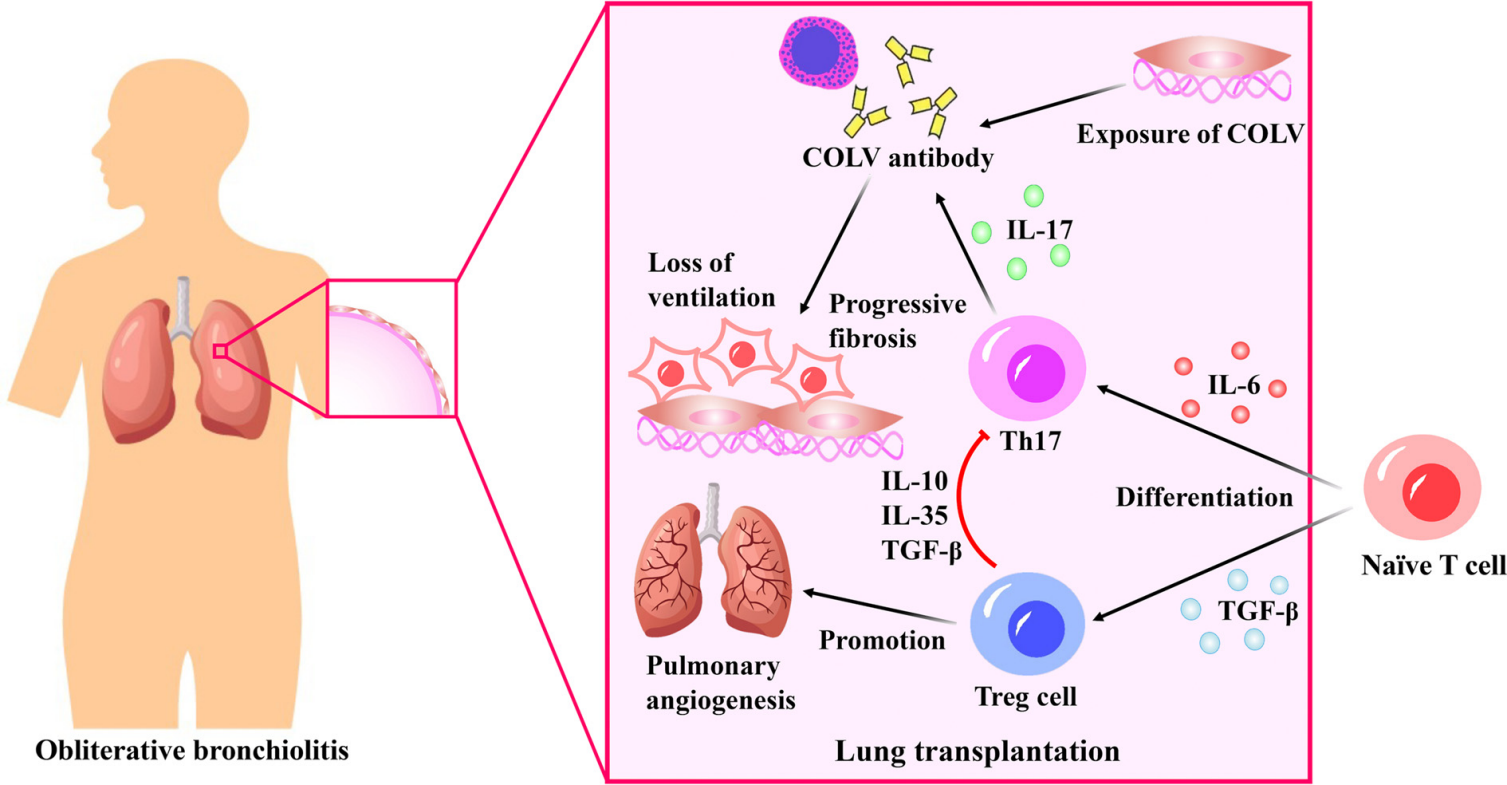
The role of regulatory T (Treg) cells following lung transplantation After lung transplantation, type V collagen (COLV) exposure leads to the production of antibodies that lead to pulmonary fibrosis and breathing difficulties. COLV antibodies are dependent on interleukin (IL)-17 produced by Th17 cells. naïve T cells will differentiate into Th17 cells in the presence of IL-6, which will further promote COLV antibody production. While TGF-β can promote the transformation of naïve T cells into Treg cells, which can produce cytokines such as IL-10, IL-35, and transforming growth factor-beta (TGF-β) to inhibit Th17 activity. In addition, Treg cells can promote pulmonary angiogenesis.

The majority of evidence suggests that the adaptive immune response in transplanted lung tissue can result in the primary risk factor for BO after lung transplantation, which is the damage caused by autoimmune mediators [276]. One of the key features of inflammatory diseases is an imbalance in the Th1/Th17 response, often accompanied by a reduction and/or alteration in regulatory Treg cells [169,279]. Recent research has demonstrated that maintaining a balance between Treg cells and Th17 cells is a potential strategy for preventing autoimmune diseases and inhibiting the development of BO after transplantation (Figure 4).

The differentiation of Treg cells and Th17 cells is dependent on the presence of TGF-β. When present in low concentrations along with IL-6, TGF-β and IL-6 induce the production of transcription factors RORγt and RORα in T cells, which activate the initial T cells to differentiate into pro-inflammatory Th17 cells. At the same time, they inhibit the expression of Foxp3, the transcription factor of Treg cells. However, in high concentrations, TGF-β can up-regulate the expression of Foxp3, resulting in the differentiation of anti-inflammatory Treg cells [280]. Moreover, TGF-β expression in Treg cells helps maintain their anti-inflammatory function following migration from the thymus [281]. Disrupting the balance of Treg/Th17 cells can cause a range of inflammatory reactions that harm the body [169]. Romagnani et al. have shown that Th17 and Treg cells come from the same precursor, and inhibiting the production of Th17 cells can promote the development of Treg cells, thus effectively preventing the occurrence of autoimmune diseases by the secretion of IL-10, IL-35 and TGF-β [282].

It was shown that lung transplant patients with BO display a systemic activation of T cells, including Th1, Th2, and Treg cells, in comparison with healthy individuals. However, in comparison to those developing BO, patients with stable BO exhibit a higher presence of Treg and Th2 cells in their blood, bronchi and alveolar lavage fluids [232]. Moreover, Treg cells have an important role in regulating pulmonary angiogenesis. D’Alessio et al. showed that pulmonary angiogenesis was significantly reduced in mice with depleted Treg cells, but the amount of angiogenesis was almost fully restored after the adoptive transfer of homologous Treg cells [283]. This provides further evidence of the involvement of Treg cells in preventing the onset of BO after lung transplantation. Once autoimmunity and alloimmunity start, they become difficult to suppress, and the absence or damage of Treg cells after transplantation leads to insufficient numbers of Treg cells in the body, which cannot exert immunosuppressive effects [284]. Meloni et al. have revealed that patients with BO have lower levels of Treg cells in their blood compared with healthy lung transplant patients, indicating defects in the Treg cell population [285]. Moreover, infection, acute rejection, or other damage can impair the function of Treg cells or convert them into other effector cells. Some studies have shown that *in vitro*-induced Treg cells rapidly lose Foxp3 expression activity after transfer to living organisms[286]. Currently, there is only one clinical trial utilizing Treg cells in the treatment of post-lung transplant rejection registered in ClinicalTrials.gov (NCT00340951), and the outcomes of this research have not yet been disclosed. Hence, utilizing Treg cells for treating BO after lung transplantation is still a major focus of research.

## Conclusion and further directions

Lung diseases can have multiple causes and result in high morbidity and mortality rates. Lung transplantation is currently the only viable treatment option for end-stage lung disease. However, the development of BO caused by chronic rejection after transplantation is the primary limitation of this procedure. Recent research on Treg cells has highlighted their potential significance in treating lung diseases.

Currently, numerous studies have indicated the involvement of Treg cells and Th17 cells in various lung diseases, including asthma, ARDS, COPD, lung cancer and pulmonary infectious diseases, etc. Treg cells and Th17 cells represent two opposing T cell subsets, with Th17 cells promoting inflammation and Treg cells playing a crucial role in maintaining self-tolerance. When the balance between Treg and Th17 cells is disrupted, inflammation may occur. Therefore, the balance of Treg and Th17 cells can be regulated by adjusting the expression of cytokines such as IL-17A, IL-6, IL-23, IL-33 and Foxp3 to achieve the goal of treating lung diseases. In the case of BO, the disease is caused by autoimmune-mediated injury caused by the adaptive immune response of the transplanted lung tissue. The balance between Treg and Th17 cells is critical in preventing autoimmune diseases and inhibiting the onset of BO after transplantation. As a result, the combined action of TGF-β and related cytokines, such as IL-2, IL-6 and IL-21, can help regulate and maintain the balance of Treg and Th17 cells, thereby suppressing the occurrence of BO.

For example, studies have shown that asthma has Th2 (Th2 cells play a central role) and non-Th2 (Th17 and Th1 cells play an important role) phenotypes [287], Treg cells exhibit a significant inhibitory effect on Th cell responses, demonstrated by Treg cells with the TIGIT phenotype selectively inhibiting Th1 and Th17 cell responses [288]. As a result, Treg cells play a crucial role in regulating conditions like asthma. Moreover, the balance between Th17 and Treg cells, represented by the Th17/Treg ratio, has a significant impact on the early onset of ARDS, making its regulation crucial for ARDS treatment [289]. Studies have indicated that transplanting Treg cells into ARDS model mice can effectively reduce pro-inflammatory cytokine levels in the alveoli [290]. Treg cells also play a vital role in ALI regression, as they can induce apoptosis of neutrophils, which helps reduce fibrocyte recruitment and ALI fibrosis [291,292]. In the context of COPD, Th17 cells produce pro-inflammatory factors like IL-17, while Treg cells release anti-inflammatory cytokines such as IL-10 and TGF-β, effectively controlling lung inflammation [293]. In NSCLC, plasma levels of IL-35 are higher in NSCLC patients compared with healthy individuals [294]. IL-35, secreted by Treg cells, exerts an immunosuppressive effect by inhibiting T-cell proliferation and function [295,296]. However, Treg cells also contribute to immune escape and tumour growth by inhibiting the host immune response through TGF-β and IL-35 [297,298]. Understanding the role of IL-35 and Treg cells in NSCLC is essential for gaining valuable insights into potential treatment approaches [299].

In terms of treatment, it was shown that Treg cells can be used in the clinical treatment of GVHD [300]. There are two primary sources of Treg cells: endogenous Treg cells in the patient’s body, which can be increased in quantity through induction, and donor-derived Treg cells, which are primarily suitable for preventing and inhibiting rejection after transplantation. Donor Treg cells can be extracted and amplified *in vitro* through selective procedures for use in patients (i.e., allogeneic use) [301]. However, this method does not apply to the treatment of autoimmune diseases.

Due to their immunomodulatory and anti-inflammatory characteristics, Treg cells play a pivotal role in managing lung diseases. Augmenting Treg cell numbers or enhancing their function can effectively inhibit inflammation and slow down the progression of conditions such as autoimmune lung diseases, asthma, ARDS, chronic obstructive pulmonary disease, cystic fibrosis, pneumonia, etc [170,182,186,237,302–304]. Conversely, for certain lung diseases like lung cancer, restraining the proliferation and differentiation of Treg cells is essential to reduce tumour burden and increase anti-tumor immune response, thus achieving therapeutic outcomes [220,305,306]. Additionally, by understanding the extent of fibrosis progression, managing the balance of Treg cells within the body, and strategically timing their deployment, it becomes possible to exert finer control over Treg cells, offering potential benefits in the management of IPF [174]. Studies have suggested that harnessing Treg cells for the treatment of lung diseases and lung transplantation represents a breakthrough in enhancing therapy and carries significant research implications. However, substantial challenges remain to be addressed, such as the need for large-scale *in vitro* cultivation, the acquisition of antigen-specific Treg cells, balancing quantity and function, and producing substantial numbers of functional Treg cells. Currently, there are three main methods. The first is *in vivo* expansion, achieved by promoting the growth of Treg cells. For instance, the use of IL-2 and TNF superfamily member 15-immunoglobulin combination or the drug RGI-2001 can increase the number of Treg cells for *in vivo* expansion [307–309]. The second method involves *in vitro* expansion. Various techniques are currently used for *in vitro* Treg cell amplification, such as cord blood-based amplification and rapamycin-based amplification [310–313]. The third method to increase the number of Treg cells is genetic engineering. The expanded polyclonal Treg cells can be genetically modified to synthesize chimeric antigen receptors or artificial cell receptors, resulting in a large number of antigen-specific Treg cells [314–316].

It was shown that the delicate balance in the human body enables minor environmental changes to trigger Treg cell responses, and modifications in the cytokine environment can impede Treg cell reconstruction, altering their efficacy [317]. Thus, in the large-scale expansion of Treg cells, the culture environment necessitates pre-treatment. The common approach involves incorporating relevant cytokines, such as IL-2 and IL-15, into the growth medium, which can sustain Treg cell stability and boost their expansion. Treatments involving rapamycin, TGF-β, and trans-retinoic acid can curb the growth of contaminated cells, resulting in higher Treg cell purity [318–320]. Additionally, donor-derived Treg cells are susceptible to interference from various endogenous factors in the treatment of rejection after transplantation, which can affect the reconstruction of these cells. This effect can be regulated through a low dose of IL-2, thereby sustaining Treg cell homeostasis [321]. In summary, pre-treatment of the culture environment is essential before *in vitro* induction to ensure smooth induction of cells without damaging Treg cells.

In conclusion, research on the Treg cells and their clinical application is rapidly developing. Numerous studies suggest that the utilization of Treg cells in the treatment of lung diseases and lung transplantation is a breakthrough in improving treatment and has great research significance. However, there are still major challenges to overcome. How to conduct large-scale in vitro culture, obtain antigen-specific Treg cells, reconcile quantity and function, and produce a large number of functional Treg cells? These issues require further investigation and improvement. As a result, utilizing Treg cells for the clinical treatment of lung diseases and lung transplantation is a hot research area with vast prospects and far-reaching significance.

## Abbreviations

ALI: acute lung injury
APC: antigen-presenting cell
ARDS: acute respiratory distress syndrome
BLIMP1: B lymphocyte-inducible maturation protein 1
BO: bronchiolitis obliterans
BOS: bronchiolitis obliterans syndrome
CAP: community-acquired pneumonia
CCL: C-C motif chemokine ligand
CCR: C-C motif chemokine receptor
CF: cystic fibrosis
CFTR: cystic fibrosis transmembrane conductance regulator
CNS: central nervous system
COLV: Type V collagen
COPD: chronic obstructive pulmonary disease
COVID-19: Coronavirus disease 2019
CTLA-4: cytotoxic T-lymphocyte-associated protein 4
CXCR: C-X-C motif chemokine receptor
DC: dendritic cell
ELX/TEZ/IVA: elexacaftor/tezacaftor/ivacaftor
GITR: glucocorticoid-induced tumor necrosis factor receptor
GITRL: glucocorticoid-induced tumour necrosis factor receptor ligand
GQD: Ge Gen Scutellaria Tang
HIF-1α: hypoxia-inducible factor 1α
HO-1: heme oxygenase 1
ICOS: inducible costimulator
IDO: indoleamine 2,3-dioxygenase
IFN-γ: interferon-γ
IL: interleukin
ILC2s: Type 2 innate lymphocytes
IPF: idiopathic pulmonary fibrosis
KGF: keratinocyte growth factor
LAG-3: lymphocyte activation gene 3
MHC-I: major histocompatibility complex class I
MP: mycoplasma pneumoniae
mTORC1: mammalian target of rapamycin complex 1
NAC: N-acetylcysteine
NK: natural killer
NRP-1: Neuropilin-1
NSCLC: non-small cell lung cancer
nTreg cell: naturally developed Treg cell
PA: *Pseudomonas aeruginosa*
PBMC: peripheral blood mononuclear cell
PD-1: programmed cell death 1
pTreg cell: peripheral original peripherally-derived Treg cell
RSV: respiratory syncytial virus
SCLC: small cell lung cancer
SPP1: secretory phosphoprotein 1
STAT: signal transducer and activator of transcription
TCR: T-cell receptor
Tfh: T follicular helper
Tfr: follicular Treg
TGF-β: transforming growth factor-beta
Th1: Type 1 T helper
Th17: Type 17 T helper
Th2: Type 2 T helper
TIM: T-cell immunoglobulin and mucin domain
TME: tumor microenvironment
Tr1 cell: Type 1 regulatory T cell
Treg: Regulatory T
tTreg cell: thymus-derived regulatory cell
VEGF: vascular endothelial growth factor
XBCQ: Xuanbai Chengqi decoction
